# MRI, unilateral NMR, macro-vision histology, texture and biochemical dataset for studying the tomato during air drying

**DOI:** 10.1016/j.dib.2026.112653

**Published:** 2026-03-16

**Authors:** Guylaine Collewet, Amidou Traore, Alexandre Leca, Sylvain Challois, Clémentine Lorho, Gauvain Pierre, Nathan Rausch de Traubenberg, Yves Diascorn, Stéphane Quellec, Caroline Garcia, Carine Le Bourvellec, Valérie Serra, Sylvie Clerjon, Marc Lahaye, Nadia Bertin, Maja Musse

**Affiliations:** a INRAE, OPAALE, 35044 Rennes, France; b INRAE, QuaPA, 63122 Saint-Genès Champanelle, France; c INRAE, Avignon Université, UMR SQPOV, 84914 Avignon, France; d INRAE, BIA, 44316 Nantes, France; e INRAE, PSH, 84914 Avignon, France

**Keywords:** Magnetic resonance imaging, Nuclear magnetic resonance, Transverse relaxation, Fruit processing, Dehydration, Fleshy fruit, *Solanum lycopersicum*

## Abstract

This dataset describes the drying process of two tomato cultivars, H1311 and Terradou, with a particular focus on the tomato pericarp. The dataset originates from two interlinked experiments. The first experiment involved continuously imaging tomato slices using an MRI scanner while they were drying. The second experiment involved analysing fresh and dried tomato slices at three different stages using MRI, unilateral NMR, macro-vision histology, tissue texture analysis and biochemical analysis. The dataset includes original and processed MRI images acquired using a 1.5 T whole-body MRI scanner, original and processed NMR signals acquired using a unilateral NMR device, macro-vision images acquired using a high-resolution C-mount digital camera and texture and biochemical analyses. These data contribute to a better understanding of the changes in the morphology, tissue microstructure and biochemical composition of the fruit during drying, and of the relationship between these phenomena and the quality of the dried product. The dataset is shared to enable extended analyses by the multidisciplinary scientific community and manufacturers interested in fruit drying.

Specifications Table

**Table:** 

Subject	Engineering & Materials science
Specific subject area	MRI and unilateral NMR (UNMR) analyses, macro-vision histology, texture and biochemical dataset related to tomato fruit drying*.*
Type of data	Original MRI Image in dicom Processed MRI images in raw format Avizo project Temporal NMR transversal echo decay signal in text Macrovision images in tiff Tables in text, cvs and xlsx
Data collection	MRI images were acquired using 1.5T scanner (Avanto, Siemens) using the SoftwareSYNGO (Siemens, version VB17) for image acquisition. MRI image processing was realized using Avizo 3D Pro (version 2021.2, FEI Company), ImageJ (1.54 g, Wayne Rasband and contributors, National Institute of Health, USA), Matlab 2019a (MathWorks, USA) ans scilab 6.1.1 for image processing*;* Unilateral NMR: Transversal signal decay were recorded using Carr Purcell Meiboom Gill (CPMG) pulse sequence on Mobile Universal Surface Explorer (MOUSE, Magritek, Germany) before being processed on Matlab 2022b (MathWorks, USA) Macro-vision: camera: Baumer VCXU31C (Baumer, sensor: Sony IMX252, Baumer SAS, France), macro imaging lens: VS technology 0513 (VS Technology Corporation, Japan), image acquisition: Baumer Camera Explorer (v3.5.2, Baumer SAS, France), image processing: FIJI (ImageJ v1.54 g, Wayne Rasband and contributors, National Institutes of Health, USA) Tissue texture: TA-plus Texture Analyzer (Lloyd Ametek, USA) controlled by Nexygen Plus software (v4.0.1.184, Lloyds Instruments, USA). Biochemical analyses: sugars and acids: absorbance measurement on SAFAS flx-Xenius XM spectrofluorimeter (SAFAS, Monaco), data acquisition on SP2000 software (v7.8.58.3, SAFAS, Monaco). Carotenoids and polyphenols: chromatography on HPLC-DAD Prominence system (Shimadzu, Japan), data acquisition and analysis on LabSolutions software (version 5.97 SP1, Shimadzu, Japan*.*
Data source location	Tomato plants were grown at UR PSH, INRAE, Avignon, France (Lat: 43.91549, Lon: 4.8794) MRI data were collected at UR OPAALE, INRAE, Rennes, France (Lat: 48.1282588; Lon: −1.7007542). Unilateral NMR data were collected at UR QuaPa, INRAE, Saint-Genès Champanelle, France (Lat: 45.7103958; Lon: 3.0146469) Macro-vision, texture and biochemical composition data were collected at UMR SQPOV, Avignon, France (Lat 43.916984, Lon 4.883684)
Data accessibility	Repository name: Recherche Data Gouv repository https://recherche.data.gouv.fr/ Data identification number: MRI related data: doi:10.57745/U495CX; UNMR related data: doi:10.57745/BXVOKG; histological, composition and texture data: doi:10.57745/NHXH7M Direct URL to data: MRI related data : https://doi.org/10.57745/U495CX; UNMR related data: https://doi.org/10.57745/BXVOKG; histological, composition and texture data: https://doi.org/10.57745/NHXH7M
Related research article	Musse M, Traore A, Leca A, Lahaye M, Lorho C, Pierre G, Rausch de Traubenberg N, Garcia C, Diascorn Y, Quellec S, Le Bourvellec C, Clerjon S, Challois S, Collewet G, Bertin N. Insights into the mechanisms involved in the evolution of the structural and physicochemical properties of tomato during air drying – a study combining MRI, unilateral NMR and conventional techniques. Food Research International, 2025. 212(10):116,385. https://doi.org/10.1016/j.foodres.2025.116385

## 1 Value of the Data

• The dataset provides a multi-scale view of tomato drying, integrating dynamic multi-scale structural changes (from MRI, histology) with water status and distribution (from MRI and UNMR) and final quality attributes (tissue texture, biochemical composition), enabling researchers to link fruit properties to drying behavior and product quality.
• The dataset includes quantitative measurements of tomato changes during drying (water and air distribution, volume shrinkage from MRI and UNMR) and final product quality (tissue texture analysis: hardness, springiness, gumminess; and biochemical composition: sugars, acids, carotenoids, polyphenols.
• The data allows for the characterization of parameters that are expected to govern the drying process, such as cell size (from histology), apparent micro-porosity (from MRI), and water compartmentalization (from $T_{2}$ relaxometry using MRI and UNMR), which are important for understanding and modeling water transport mechanisms.
• The dataset is valuable for developing and validating mathematical models of heat and mass transfer during fruit drying, as it provides actual data on tissue structure, water content evolution and shrinkage.
• The dataset is useful for NMR and MRI scientists interested in interpretation of changes in transverse relaxation parameters during plant tissue processing.
• The raw and processed MRI and UNMR data may serve as a test case for NMR/MRI scientists developing signal and image processing methods, particularly for multi-exponential relaxation analysis.
• The dataset is useful for food scientists, biologists, and the food industry interested in optimizing drying processes, selecting cultivars better suited for drying, and understanding how processing conditions affect final product quality and nutritional value.

## 2 Background

**Fig. 1:**
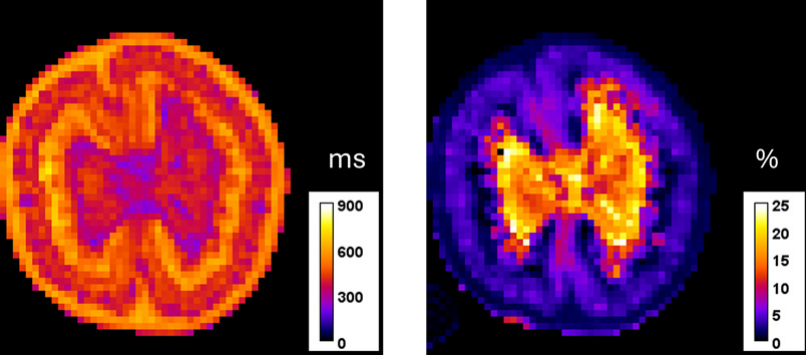
Example of MRI quantitative measurements on a fresh tomato: on the left mono-exponential $T_{2}$ relaxation time in ms, on the right micro-porosity map in %.
• Unilateral NMR was evaluated as a potential low-cost, portable tool for rapid, non-destructive quality assessment. UNMR depth profiles provide a one-dimensional measurement of signal intensity (proportional to water amount) as a function of depth through the tomato slice. This allows to track how water is lost from different depths during drying. UNMR also provides $T_{2}$ distributions which reveal different water pools and how they evolve during drying.
• Histology, tissue texture and biochemical analyses provide data on cell structure, mechanical properties, and nutritional composition needed to characterize fruits and to interpret and validate the information obtained from the non-destructive MRI and UNMR methods.

Drying is an essential preservation method for highly perishable fleshy fruits, but it also affects their texture and nutritional value. The dehydration rate, texture and nutritional quality of dried fruit are influenced by the drying method used and the fruit’s compositional and structural properties, including the organization of water at tissue and subcellular levels. Understanding these complex phenomena helps to optimize the drying process for different tomato varieties, resulting in high-quality, energy-efficient dried products. To facilitate this, it is necessary to gather data describing changes in the fruit's morphology, tissue microstructure, biochemical composition and quality indicators, such as tissue texture. MRI, unilateral NMR (UNMR), macro-vision histology, tissue texture and biochemical dataset was designed to investigate the changes in tissue structure during drying, particularly in water distribution and compartmentalization, and their link to the final texture and nutritional quality of the dried product. The purpose of combining these techniques was to obtain a comprehensive picture of the drying process:

• MRI was used to provide a spatially resolved, non-destructive view of water status and distribution (via $T_{2}$ measurements) and tissue micro-porosity within the whole tomato slice. This allows for the study of heterogeneities, such as the difference in drying behavior between the outer and inner pericarp. Example of quantitative $T_{2}$ and micro-porosity maps are given in Fig. 1. MRI also provided information on the evolution of fruit volume during drying.

This dataset is related to the research paper “Growth kinetics, spatialization and quality of potato tubers monitored in situ by MRI - long-term effects of water stress” [1] and allow for advanced analysis by food scientists, biologists, and manufacturers with an interest in the fruit drying process. It also supplies data for NMR/MRI scientists interested in better understanding changes in transverse relaxation parameters during plant tissue dehydration and in developing new signal and image processing methods.

## 3 Data Description

### 3.1 Introduction

This data set covers the original and processed MRI images and UNMR signals describing the drying process of two tomato cultivars, H1311 and Terradou, as well as histological, textural and biochemical data measured on tomato samples. Tomato slices with the internal tissues (placenta, seeds and jelly) removed, leaving only the pericarp, were analyzed. Two different trials, corresponding to two experimental approaches, were led. They are named Experiments A and B hereafter. Although the MRI samples names are the same for Experiment A and B, they do not correspond to the same tomato batches.

The dataset (https://doi.org/10.57745/U495CX) related to MRI analyses contains 15 compressed files and 9 tables (Excel files) for experiments A and B.

The dataset (https://doi.org/10.57745/BXVOKG) related to UNMR analyses of experiment B, contains two compressed files, for T2 and profiles, along with a data_Description describing the file structure hierarchy of each archive.

The dataset (https://doi.org/10.57745/NHXH7M) related to histological, textural and biochemical analyses of experiment B, contains in the first layer 2 zip archives, 1 table (csv file), 1 RStudio statistical data analysis file (Rmd file) and its attached ‘knitted’ output (pdf file), and 1 documentation file (md file). In total (including the subfolders) the data set contains 23 tables (csv and Excel files), 207 images (tif files), and one R Markdown file, one PDF file, and one documentation markdown file.

### 3.2 Experiment A

One batch was used for this experiment, named batch A1. MRI images were acquired continuously during the drying of the tomato slices in a temperature-controlled chamber placed inside the imager. Eight tomatoes per cultivar were analyzed. The samples were labeled H01 to H08 for the cultivar H1311, and T01 to T08 for the cultivar Terradou. Two replicates were analyzed, each of them including four ripe tomatoes of each cultivar: H01, T01, H03, T03, H05, T05, H07 and T07 for the first replicate (ExpA_replicat1), H02, T02, H04, T04, H06, T06, H08 and T08 for the second replicate (ExpA_replicat2). Mono-exponential $T_{2}$ maps and pericarp volumes were estimated.

### 3.3 Experiment B

MRI, UNMR, and histological, textural and biochemical analyses of fresh and dried tomato slices were performed. The fresh tomato slices to be used for the different measurement methods were dried to three predefined drying degrees (DD1 (90% of water content), DD2 (85% of water content), DD3 (50% of water content)) under similar conditions before analyses.

MRI, UNMR and the set of histological, textural and biochemical analyses were done on three different batches, respectively numbered B1, B2, B3.

Fifteen tomatoes per cultivar of batch B1 were analyzed by MRI. The samples were labeled H01 to H15 for H1311, and T01 to T15 for Terradou. MRI was performed on all fresh fruits. Following this initial MRI analysis, slices with centers corresponding to those of the virtual equatorial tomato slices in the MRI images were cut and dried. For each cultivar, numbers 01 to 05 were dried to the drying degree DD3, 06 to 10 to DD2 and 11 to 15 to DD1. Mono and tri exponential $T_{2}\;$maps, $T_{2}^{*}\;$maps, μ-porosity maps and pericarp volumes were estimated.

At least 3 tomatoes per cultivar of batch B2 were analyzed per drying degree by UNMR. The profile analysis were labeled P_H(T)_DDx(FRESH)_Ey where H or T stand for H13311 or TERRADOU, FRESH for fresh sample and x represent the drying delays, 2 for 7 h and 3 for 16 or 18 h and y represents the sample number. In the file names, mm and dd represent the month and day of the experiment.

The samples used for T2 analysis were labeled similarly with T2 prefix in place of P . Different tomatoes were used for analyses on fresh and dried slices.

Batch B3 was composed of five tomatoes (numbered 01 to 05) of each cultivar and drying state. Three more H1311 tomatoes (numbered 06, 07, 08) were used in biochemical analyses to strengthen data. The texture analysis was performed before preparation for biochemical composition analyses. However, no texture analysis was performed on samples dedicated to histology.

### 3.4 Files format

#### 3.4.1 MRI

MRI images are in DICOM format that consists of a header with acquisition parameters and image data sets packed into a single *.dcm file.

Processed MRI images, mono and tri exponential $T_{2}\;$maps, $T_{2}^{*}$ maps and µ-porosity maps, are stored in « raw » format : only the pixel values are stored (no header) in float precision. To open them using image J, use the following parameters for “Import raw”:

To open them using Matlab, use the following code:

fd=fopen(name_of_the_image,’rb’);

image=reshape(fread(fd,192×192,’float32’),192,192) :

fclose(fd);

Files representing the masks that distinguish tomatoes from the background are images in PNG format.

Regions of interest corresponding to the whole pericarp are stored in a zip format readable with ImageJ software.

Each Avizo analysis is stored in a .hx file combined with a folder of the same name. They can be opened using Avizo software.

The statistical results are stored in text format (.tab)

#### 3.4.2 UNMR

All the UNMR dataset are in text (.dat) format, or in csv and xlsx (Excel Files).

#### 3.4.3 Histology

All results are gathered as ASCII text in csv files. All macrovision images are in tiff format (.tif extension).

#### 3.4.4 Tissue texture

All data are gathered as ASCII text in a single csv file. They were copied directly (without modification) from the raw data (bch file extension), proprietary of Nexygen software.

#### 3.4.5 Chemical composition

All data are gathered as ASCII text or extended text in csv or Excel (.xlsx extension) files.

### 3.5 Files description

#### 3.5.1 Experiment A

ExpA_MRI_1_images_FR-TSE.zip and ExpA_MRI_1_images_MSE.zip contain the MRI images acquired using the protocols FR-TSE and MSE respectively. They are provided at : doi:10.57745/1VKL0X and doi:10.57745/ZHBAHB respectively.

There are two sets of images, one for ExpA_replicat1, the other one for ExpA_replicat2. For each set, 15 different time points were acquired resulting in 15 different folders names respectively FR-TSE_time_xx and MSE_time_xx with xx varying from 01 to 15.

The position of each tomato on the images are as follows:

In the file ExpA_MRI_2_cartographies_T2_mono.zip, provided at doi:10.57745/WPYN2K, both masks used for the $T_{2}$ relaxation maps estimations as well as mono exponential $A_{0}$ and $T_{2}$ maps can be found. The masks are in the folders “mask_for_T2_mono_cartographies” which is divided in two sub-folders for each set of tomatoes ExpA_replicat1 and ExpA_replicat2.

For the $A_{0}$ and $T_{2}$ maps, in the subfolder “regrouped per type”, the tomatoes have been regrouped by cultivar, H1311 and Terradou, and in each subfolder, $T_{2}$ maps, $A_{0}$ maps and the corresponding masks are found, each for each time from 1 to 15. In these maps regrouped per type, the tomatoes are in these positions:

ExpA_MRI_3_avizo_analysis.zip, provided at doi:10.57745/IM4ZAH, contains two main folders (one for ExpA_replicat1 the other one for ExpA_replicat2). In each folder Avizo files are given for times from 1 to 15.

ExpA_MRI_pericarp_T2_per_tomato_replicat1.tab, provided at doi:10.57745/SAFI9W, and ExpA_MRI_pericarp_T2_per_tomato_replicat2. tab, provided at doi:10.57745/JUZ4Q1, contain the evolution of the mean $T_{2}$ values of each tomato pericarp regarding time (first column) for ExpA_replicat1 and ExpA_replicat2 respectively.

ExpA_MRI_pericarp_volume_per_tomato_replicat1. tab, provided at doi:10.57745/VRDROC, and ExpA_MRI_pericarp_volume_per_tomato_replicat2. tab, provided at 10.57745/1RKGVZ, contains the evolution of volumes of each tomato slice regarding time (first column) for ExpA_replicat1 and ExpA_replicat2 respectively.

ExpA_MRI_T2_mono_profile_per_tomato. tab, provided at doi:10.57745/LBBOY9, contains the evolution of $T_{2}$ mean values in function of the distance to the cuticle for ExpA_replicat1 and ExpA_replicat2. The first column indicates the cultivar, the second the tomato, the third the distance to the skin and the next columns are for the $T_{2}$ mean values at each successive time. The distance is from 1 to 10 mm, spaced by 1 mm, so that the measurements for each tomato correspond to 10 lines, one for each distance.

#### 3.5.2 Experiment B

##### 3.5.2.1 MRI

ExpB_MRI_1_images_FR-TSE.zip, provided at doi:10.57745/WGTBAC, ExpB_MRI_1_images_MGE.zip, provided at doi:10.57745/JZ6TYS, and ExpB_MRI_1_images_MSE.zip, provided at doi:10.57745/S1TP5P, contain the original MRI images acquired with the protocols FR-TSE, MGE and MSE respectively. Acquisitions were made separately for tomatoes H1311 and Terradou which results in two folders, themselves divided in two folders for fresh (FRESH) and dried (DD1_DD2_DD3) stages. Each FRESH folder is subdivided in three folders for tomatoes 01 to 05, 06 to 10, and 11 to 15.

The position of each tomatoes on the images of fresh tomatoes are as follows for H1311:

The position of each tomato on the images of fresh tomatoes are as follows for Terradou:

Each DD1_DD2_DD3 folder is divided in two folders, one for tomatoes 01, 02, 03, 06, 07, 08, 11 and 12, the other one for tomatoes 04, 05, 09, 10, 13, 14 and 15. The position of each tomato on the images are as follows:

The processed images for experiment B are stored in the same order as the MRI images. ExpB_MRI_2–1_cartographies_T2_mono.zip, provided at doi:10.57745/PD5OWA, contains the $A_{0}$,$\;T_{2}$ mono-exponential maps. ExpB_MRI_2–1_cartographies_T2_tri.zip, provided at doi:10.57745/Z10NDY, contains the $A_{0}$,$\;T_{2}$ tri-exponential maps. The amplitudes expressed in percentage of the total amplitudes are also provided (pct_A0 folder). The maps for the shortest $T_{2}$ component are not provided since they could not be properly estimated. ExpB_MRI_2–3_cartographies_T2_star.zip, provided at doi:10.57745/ISN2LV, contains the $T_{2}^{*}$ maps. ExpB_MRI_2–4_cartographies_porosity.zip, provided at doi:10.57745/ICBMSA, contains the μ-porosity maps. ExpB_MRI_2–5_cartographies_mask_for_T2_T2_star_porosity.zip, provided at persistentId=doi:10.57745/Q3KGKH, contains the masks used for the estimation of mono, tri $T_{2}$ and $T_{2}^{*}$ star.

ExpB_MRI_3_cartographies_ROIs_pericarp.zip, provided at doi:10.57745/75ZHUH, contains the Region of interest (ROI) for FRESH, DD1 and DD2. The number of the concerned tomatoes are indicated in the names of the zip files (e.g. for DD1 ROI_H_13_14_15.zip). These ROIs were used to compute the mean values on mono, tri-exponential $T_{2}$ and μ-porosity maps. ExpB_MRI_4_cartographies_mask_for_profiles.zip, provided at doi:10.57745/T01N8G, contains the mask used for the computation of the mean values in function of the distance to the cuticle for FRESH, DD1 and DD2. In the name of the files the number of the concerned tomatoes are indicated (e.g. for DD1 mask_H_13_14_15.PNG)

ExpB_MRI_5_avizo_analysis.zip, provided at doi:10.57745/RJ7WRT, contains two main folders for each type of tomato. The files are given for fresh tomatoes only.

ExpB_MRI_data_1_fresh. tab, provided at doi:10.57745/RWMOBM, contains the following data for the fresh tomatoes: tomato, drying degree, total volume of the tomato fruits, total mass of the tomato fruits, volume of the tomato slices (containing pericarp and cuticle), mass of the tomato slices, water content of the tomato slices, mono and tri-exponential $T_{2}$ means, μ-porosity means. ExpB_MRI_data_1_dried. tab, provided at doi:10.57745/X6AOSX, contains the following data for the dried tomatoes: tomato, drying degree, drying time in h, mass of the slices, volume of the slices, water content, mono and tri-exponential $T_{2}\;$means, μ-porosity means. ExpB_MRI_T2_mono_profile_per_tomato. tab, provided at doi:10.57745/N7A9FB, contains the mono-exponential $T_{2}$ mean values in function of the distance to the cuticle for each tomato and each drying degree (including fresh).

ExpB_MRI_porosity_profile_per_tomato.xlsx, provided at doi:10.57745/GZIS14, contains the μ-porosity mean values in function of the distance to the cuticle for each tomato and each drying degree (including fresh). For these two files the information is as follows: cultivar, tomato, drying degree (including fresh), measurements at increasing values of distance to the cuticle.

##### 3.5.2.2 UNMR

###### 3.5.2.2.1 Profiles

For both cultivars, raw and processed NMR depth profile data are in the compressed (win.rar RARLab® GmbH, Germany) file named Experiment_B_UNMR_PROFILES.rar, provided at doi:10.57745/P7GERH. The decompression of this file will provide the folder tree structure hierarchy beginning with the cultivar folder, P_H1311 and P_TERRADOU respectively. Each of them contains sample folders named P_H(T)_DDx(FRESH)_Ey where H or T stand for H13311 or TERRADOU, FRESH for fresh sample and x represent the drying delays, 2 for 7 h (DD2) and 3 for 16 or 18 h (DD3) and y represents the sample number.

Each sample folder contains the raw data files as provided by the spectrometer (Propa®, Magritek, Aachen, GERMANY). In the file names, the two numbers _mm_dd represent respectively the month and day of the experiment.

These files are :

- acqu.par : text file containing the summary of the acquisition parameters
- XXX_mm_dd-decays.dat (XXX prefix represent the sample name): text file containing the 80 depths of 256-echo decays. The first colon in this file represents the sampling time 0.092 ms to 23.552 ms.
- XXX_mm_dd.dat (XXX prefix represents the sample name) is a text file containing the reconstructed profiles from the decays in XXX-decays.dat. There are 5 colons in this file. The first colon represents the 80 depths, the second one represents the signal amplitude obtained by averaging the 16 first echoes of the decay. In the third one, 64 first echoes were averaged and in the fourth one, the 256 echoes were averaged. These different parameters of contrast (parContrast1 = "1,16″, parContrast2 = "1,64″, parContrast3 = "1,256″) can be found in the acqu.par file. The fifth colon represent the contrast ratio between the initial amplitude (sum of the 64 first echoes and those of the remaining 192 echoes as specified by parAmpIni = "1,64″ and parAmpFin = "64,256" in the acqu.par file. It should be noted that the contrast3 averaging the full 256 echoes was used in the research article.

###### 3.5.2.2.2 T2

For both cultivars, raw and processed CMPG decays data for T2 measurement are in the compressed file named Experiment_B_UNMR_T2.rar, provided at doi:10.57745/P81YNB. The decompression of this file will provide the folder tree structure hierarchy beginning with the cultivar folder, T2_H1311 and T2_TERRADOU respectively. Each of them contains sample folders named T2**_**H(T)_DDx(FRESH)_Ey where H or T stand for H13311 or TERRADOU, FRESH for fresh sample and x represent the drying delays, 2 for 7 h (DD2) and 3 for 16 or 18 h (DD3) and y represents the sample number.

Each sample folder contains subfolders named as T2_p_XXmm, where XX mm indicates the depth(s) where T2 measurement was performed. In these suborders, the acquisition parameters are summarized in the acqu.par file. The 3-colon file data.csv contains the acquired transversal decay data where the first one is the sampling time in ms, the second and the third are real and imaginary echo amplitudes. The decay plot is presented in the data.png file. As the receiver was phased (parameter rxPhase = 236) to obtain flat imaginary part, only the real part was used in the research article. To ease the analysis, each data.csv file was renamed conveniently and put in the folder DECAYS_NNLS_FIT_Results_H1311(TERRADOU). The result of NNLS fit of each decay is also provides as a matlab (Matlab®, The MathWorks, Inc) .fig file along with a H(T)_NNLS_Results_T2Distribution.xls excel file containing the full summary of the T2 distribution used in the research article.

##### 3.5.2.3 Histology

Seven (7) files are gathered in the Zip archive ExpB_Destructive_6_Histology.zip.

The three (3) CSV files contain the size analysis results.

ExpB_Destructive_6_Histology__Cell_Size_Total.csv gathers all the cell size analysis performed in experiment B.

ExpB_Destructive_6_Histology__Cell_Size_Gradient_H1311.csv gathers the results of cell size gradient between the cuticle and the pericarp measured on a H1311 tomato slice.

ExpB_Destructive_6_Histology__Cell_Size_Gradient_Terradou.csv gathers the results of cell size gradient between the cuticle and the pericarp measured on a Terradou tomato slice.

The Data file columns are as follows:

- First column [no name]: object number, here each cell
- Cultivar: tomato cultivar (only for ExpB_Destructive_6_Histology__Cell_Size_Total.csv)
- State: fruit trying state (only for ExpB_Destructive_6_Histology__Cell_Size_Total.csv)
- No: individual fruit ID (only for ExpB_Destructive_6_Histology__Cell_Size_Total.csv)
- Cell: aspect of the cells, "intact" if they look undamaged, "hollow" if they look cut and emptied from their cellular material (only for ExpB_Destructive_6_Histology__Cell_Size_Total.csv)
- Label: string label (generally file name) of the image containing the current cell
- Area: Area of the measured cell in µm²
- X: X-coordinate of the center of the object (centroid)
- Y: Y-coordinate of the center of the object (centroid)
- Perim.: Perimeter (length of the outside boundary of the cell) in µm
- Major: primary axis of the ellipse fitting best to the cell in µm
- Minor: secondary axis of the ellipse fitting best to the cell in µm
- Angle: angle between the primary axis of the fit ellipse and the X-axis of the image in °
- Circ.: Circularity (shape descriptor) of the cell, 0 is a flat line, 1 is a perfect circle
- Feret: Feret diameter (longest distance between any two points of the cell) in µm
- Skew: Skewness (3rd order moment about the mean)
- %Area: Area fraction of the cell over the whole image
- FeretX: starting X-coordinate of the Feret diameter
- FeretY: starting Y-coordinate of the Feret diameter
- FeretAngle: angle between the Feret diameter and the X-axis of the image in °
- MinFeret: minimum distance between two points of the cell boundary in µm
- AR: Aspect Ratio of the cell over the best fitting ellipse (Major/Minor)
- Round: Roundness (shape descriptor), 0 is fully broken boundary, 1 is perfectly round and smooth cell
- Solidity: Solidity (shape descriptor) of the cell, i.e. area over convex area of the cell

The two (2) TIF files are the cell size gradient images corresponding to the cell size gradient CSV files bearing the same name.

The two (2) Zip archives contain images and individual file results (gathered in the CSV files described above).

All sub-archives files are named in the form Cultivar_State_Individual Fruit ID.zip and contain:

- a 'Not Used' folder in which the images not good enough for analysis are stored
- a 'Used' folder in which all the images used in the analysis are stored
- 1 to 3 files named in the form Results_Macro_Cultivar_State_Individual Fruit ID_specific information.zip
- 1 or more TIF files named in the form Scale_Cultivar_State_Individual Fruit ID_name of the image.tif. These images were used a size measurement scale for image analysis.

##### 3.5.2.4 Tissue texture

The file containing all the Texture data is named: ExpB_Destructive_8_Texture.csv

Texture Profile Analysis (TPA) consists in 5 consecutive steps:

1. probe moves downwards to compress the sample until 30% deformation
2. probe moves upwards to 1 mm above the sample initial height
3. probe is still for 10 s
4. probe moves downward to compress the sample until 30% deformation
5. probe moves upwards to 1 mm above the sample initial height

The data file columns are as follows:

- SamplePassed: returns VRAI(‘TRUE’) if the test is valid, otherwise returns FAUX(‘FALSE’)
- Year: sample harvest/processing year (YYYY)
- Cultivar: tomato cultivar
- State: fruit drying state
- Num_Fruit: fruit number corresponding to individual fruit ID
- WC_real: actual Water Content (%) as calculated after drying based on the sample weight and dry matter
- Probe_Direction: checking the direction of the probe, should always be 'Compression' for this experiment
- Preload: preload threshold in N
- Preload_Velocity: preload velocity in mm/s
- Timestamp: timestamp in DD/MM/YYYY hh:mm
- User: empty column
- Software Version: checking the version of the software
- Height: sample height in mm as estimated by the position of the probe upon preload
- Max Load: maximum load during the test in N
- Machine Extension at Max Load: position of the probe at max load in mm
- Extension from Preload at Max Load: displacement of the sample from its top in mm
- Extension ratio at Max Load: (Extension from Preload at Max Load)/(Height)
- Extension % at Max Load: Extension from Preload at Max Load)/(Height) in %
- Max Extension Load: load at maximum extension in N
- Maximum Extension from Preload: maximum displacement of the probe in mm
- Deformation percentage at maximum extension:
- Adhesive force: adhesiveness of the sample calculated as the area under the curve of the first negative peak (step 2) in N.s
- Hardness1: highest load at 1st peak (step 1) in N
- Gumminess: Hardness*((area under the 2nd positive peak at steps 4 & 5)/(area under the 1st positive peak at steps 1 & 2)) in N
- Springiness: 100*(probe displacement at step 2)/(probe displacement at step 1) in %
- Firmness: maximum load measurement during the whole test in N
- Hardness2: highest load at 2nd peak (step 1) in N
- Hardness: maximum load measured between step 1 and step 2 in N
- Toughness: maximum load measurement during the whole test in Nvi. Chemical composition

##### 3.5.2.5 Chemical composition

Four (4) files contain the Chemical composition data and are gathered in Zip archive ExpB_Destructive_7_Composition.zip. Filenames are given in each sub-section.

###### 3.5.2.5.1 Dry matter

The file containing Dry Matter data is named: 2023_IRMPROSE_Results_DryMatter.xlsx. The file contains two (2) tables.

Table 1 Columns are as follows:

- Sample: sample code name (unique identifier in the form of *Project_Year_CV_State_Individual Fruit ID*)
- Project: name of the project
- Year: sample analysis year (YYYY)
- Cultivar: tomato cultivar
- State: fruit drying state
- Rep: fruit number corresponding to individual fruit ID
- DM %: dry matter in percent (%)

Table 2 Columns are as follows:

- State: fruit drying state
- Cultivar: tomato cultivar
- N°tomato: fruit number corresponding to individual fruit ID
- mass (g): weight of the sample in g
- n°sample: sample number
- DM %: dry matter in percent (%)

###### 3.5.2.5.2 Sugars and acids

The file containing Sugars and Acids data is named: 2023_IRMPROSE_Results_SugarsAcids.xlsx. The file contains two (2) sheets:

a) Calculations sheet

The primary file header (rows 1–20) describe the calibration for each molecule (row 4)
The results table starts row 22:
- Nom: row/tank number
- Sample: sample code name (unique identifier in the form of Project_Year_CV_State_Individual Fruit ID)
- Mass (g): weight of the sample in g
- Water volume (ml): water dilution volume in ml
- Dilution: dilution ratio (repeated for each molecule)
- OD (10 min): optical density measured after 10 min (repeated for each molecule)
- Conc (g/l): concentration estimated from the calibration curve in g/l (repeated for each molecule)
- Conc (g/kg): concentration estimated from the calibration curve in g/kg (repeated for each molecule)

b) DM sheet

This file contains 2 tables:

The first table is reported from Calculations and Dry Matter file:

- Sample: sample code name (unique identifier in the form of Project_Year_CV_State_Individual Fruit ID)
- columns 2–5: concentration of the corresponding molecule (column header) in g/kg
- DM %: sample dry matter in percent (%)

The second table returns a calculation of sugars and acids concentration over sample dry matter:

- Sample: sample code name (unique identifier in the form of Project_Year_CV_State_Individual Fruit ID)
- columns 2–5: concentration of the corresponding molecule (column header) over sample dry matter in g/kg
- Average G: averaged concentration of Glucose over sample dry matter in g/kg
- Average F: averaged concentration of Fructose over sample dry matter in g/kg
- Average CA: averaged concentration of Citric acid over sample dry matter in g/kg
- Average MA: averaged concentration of Malic acid over sample dry matter in g/kg

###### 3.5.2.5.3 Carotenoids

The file containing Dry Matter data is named: 2023_IRMPROSE_Results_Carotenoids.xlsx. The primary file header (rows 1–10) contains information on the standard solution

The secondary file header (rows 13–16) describe the two (2) models used : Apocarotenal/Beta-Carotene, and Apocarotenal/Lycopene

The results table starts row 18:

- No: Row number
- Sample: sample code name (unique identifier in the form of *Project_Year_CV_State_Individual Fruit ID*)
- Project: name of the project
- Year: sample analysis year (YYYY)
- CV: tomato cultivar
- DR: fruit drying state
- Rep: fruit number corresponding to individual fruit ID
- Test Weight: sample weight in mg
- Peak Area (columns 9–14): chromatogram area of the peak of the corresponding molecule (column header) at the given wavelength
- [C] FM (columns 15–19): concentration of the corresponding molecule (column header) for fresh material in µg/g
- [C] DM (columns 20–25): concentration of the corresponding molecule (column header) over sample dry matter in µg/g

###### 3.5.2.5.4 Polyphenols

The file containing Dry Matter data is named: 2023_IRMPROSE_Results_Polyphenols.xlsx. The file contains two (2) sheets:

a) Results in mg per g DM sheet

○ Sample: sample code name (unique identifier in the form of *Project_Year_CV_State_Individual Fruit ID*)
○ Project: name of the project
○ Year: sample analysis year (YYYY)
○ CV: tomato cultivar
○ DR: fruit drying state
○ Rep: fruit number corresponding to individual fruit ID

column 7–13: concentration of the corresponding molecule (column header) in mg/g

b) Pivot Table sheet

This is an automatic MS Excel pivot table (French language) resulting in mean values (row 2–13) and standard deviations (row 19–30) for each cultivar × drying state ×polyphenol

## 4 Experimental Design, Materials and Methods

### 4.1 Fruits

Tomato (*Solanum lycopersicum*) plants were grown under semi-controlled conditions in a greenhouse. Tomato seeds of two industry-type (determinate) cultivars of *Solanum lycopersicum* (H1311 and Terradou) were sown in plug trays in a climatic plant growth chamber with 70% relative air humidity, 120 µmol m^-2^ s^-1^ photosynthetic photon flux intensity, 14 h artificial daylight, and day–night temperatures set at 22–17 °C. Following germination, seedlings were transferred to a glasshouse and transplanted individually into 0.3-L pots, then, at the third true leaf stage, into 7.5-L pots filled with compost substrate made of 90% organic matter (2/3 frozen black peat moss, 1/3 peat moss) with a 80% water retention capacity (Potgrond h70 047, Klasmann-Deilmann France).

The day-night temperature setup was 20–18 °C, with the shadow screen deployed when the temperature exceeded 32 °C. The plants were irrigated with a nutrient solution (Liquoplant Rose, 4/1000 dilution, Plantin, Courthézon, France) supplied by two drippers (drip flow of 2 l/h) per pot. Irrigation was monitored according to ETP (every 0.2 mm) during the daylight period, with an average of 2 l per plant per day during the fruit growth period. The duration of each irrigation was periodically revised to match 100% replacement of plant evapotranspiration. The electrical conductivity of the nutrient solution ranged from 2.0 to 3.5 throughout the crop cycle, with a pH of around 6.0. Flowers were hand-pollinated three times a week.

Soil water content was tracked using probes (five random plants per cultivar) inserted below the soil surface (EC-5 Soil Moisture Sensor, Decagon). The average volumetric water content was approximately 45%, with a soil water potential of around −0.1 MPa.

Tomatoes were harvested at the red ripe stage for each cultivar and sent to INRAE Rennes (France). Harvested fruits were stored at 4 °C until analysis was performed a few days after delivery.

### 4.2 Sample preparation and drying process

#### 4.2.1 Experiment A

Central slices 24 mm thick were cut from the equatorial region perpendicular to the pedicle axis, and the core, locular tissue and seeds were removed, leaving only the pericarp and cuticle. Eight of the prepared fresh slices (four from each cultivar) were analyzed simultaneously. They were positioned on mesh grids, raised by 10 mm to allow uniform drying inside the MRI device. The grid and tomato slices were together placed in the temperature-controlled chamber, which provided a constant airflow (∼1 m/s) at 45±1 °C. Continuous MRI acquisitions were performed over approximately 15 h.

#### 4.2.2 Experiment B

Following the initial MRI analysis on the central section of 15 entire fresh tomatoes, 24 mm-thick slices with centers corresponding to those of the virtual equatorial tomato slices in the MRI images were cut and prepared as described for Experiment A for drying (5 tomatoes per drying degree). For UNMR and histology, texture and biochemical analyses, different tomatoes were used for measurements of fresh and dried slices, the later prepared according to protocol described above. For the unilateral NMR measurements, slices were 20 mm thick, due to the experimental constraints.

Air drying of the tomato slices was performed at 45±1 °C using comparable ventilated ovens in the different laboratories (Froilabo AP120, France for Batches B1 and B2, Memmert UF110, Memmert, Germany, for Batch B3). The slices were placed on a 10 mm mesh grid, raised by 10 mm (as in Experiment A) and were left to dry for the time required to reach three approximate target relative water content (WC) values determined in preliminary experiments: 1) 90% for drying degree 1 (DD1); 2) 85%, for drying degree 2 (DD2); and 3) 50% for drying degree 3 (DD3). After each drying step, the tomato slices were placed in at 4 °C for 15 min for thermal rebalancing. They were wrapped in plastic film and left for at least 12 h at 4 °C prior to analysis in order to stabilize the tissues after dehydration.

#### 4.2.3 Water content

Water content, WC, expressed as a percentage of fresh weight for each tomato slice, was computed following each analysis in Experiments A and B using the following equation: WC=100(FW-DW)/FW where FW and DW represent, respectively, the fresh and dry weights of the tomato slices.

DW was determined by freeze drying (Experiment A) or oven drying at 70 °C for 72 h (Experiment B, Batches 1 and 3) or at 105 °C for 12 h (Experiment B, Batch 2).

### 4.3 MRI

#### 4.3.1 Acquisition protocols

MRI measurements were performed on a 1.5 T imager (Magnetom Avanto, Siemens, Erlangen, Germany).

2D MRI experiments were performed with the following geometric parameters: coronal sections in the middle of tomato slices (Experiments A and B) and transverse sections in the middle of entire tomatoes (Experiment B) were imaged with a imaging slice thickness of 5 mm, a 192×192 pixel matrix, and a 192×192 mm² FOV. The imaging protocols were:

Experiments A and B: multi spin echo (MSE) sequence to measure $T_{2}$ maps with 512 echoes, where the first echo (TE) was equal to an inter-echo spacing (ΔTE) of 6.5 ms. Pixel bandwidth was 290 Hz/pixel, number of averages (NA) was 2 and repetition time (TR) was fixed at 20 s to prevent T_1_-weighting.

Experiment B: multi gradient echo (MGE) sequence to measure $T_{2}^{*}$ maps, with 12 echoes (first echo time TE1 = 2.27 ms and ΔTE = 1.58 ms), TR =5 s, BW = 930 Hz/pixel, flip angle = 90°, and 2 NA.

In both Experiments A and B, 3D morphological images with a spatial resolution of 0.8 × 0.8 × 0.8 mm^3^ were acquired using a fast recovery turbo spin echo (FR-TSE) sequence with the following parameters: 256×256×80(96) pixel matrix for fresh tomatoes and 256×256×44 pixel matrix for dried tomato slices, TR = 140 ms, effective TE = 28 ms, ΔTE = 9.2 ms, BW = 199 Hz/pixel, Turbo Factor = 6, 42% interpolation in the imaging slice direction and 3 NA.

In Experiment 2, MRI measurements were performed at 10 ± 1 °C, regulated by the temperature-controlled MRI device.

#### 4.3.2 Image processing

Transverse relaxation times $T_{2}$ were estimated using MSE images acquired at different echo times $t_{i}.$ The signal ${s_{MSE}}_{i}$ in each voxel was modelled as the sum of exponential decays of M components:

$${s_{MSE}}_{i}=\sum_{j=1}^{M}A_{j}e^{-t_{i}/T_{2j}}$$

where $T_{2j}$ and $A_{j}$ are respectively the transverse relaxation time $T_{2}$ of component j and its associated amplitude (conventionally, components are numbered from the shortest to longest $T_{2}$). $T_{2j}$ and $A_{j}\;$were calculated for each voxel using the method described in [2], for tri-exponential (M=3).In this instance, the calculation of the component with the shortest $T_{2}$ appears to have been compromised by the presence of acquisition artifacts; this component was therefore excluded from our analysis.

Apparent µ-porosity maps were computed following the method used in[3], which uses both mono-exponential (M=1) $T_{2}$ and $T_{2}^{*}$ values. $T_{2}^{*}$ estimation was done using MGE images and the following mono-exponential decay model for the signal ${s_{MGE}}_{i}$:

$${s_{MGE}}_{i}=\;A_{0}^{*}e^{-t_{i}/T_{2}^{*}}$$

Semi-automatic segmentation was performed on 3D FR-TSE images using Avizo software to enable estimation of slice volumes.

For fresh tomatoes of Experiment B, additional preliminary processing steps were necessary because the fruit was scanned in its entirety. First, 30 central slices of each fresh tomato were selected, corresponding to the thickness of the fruit slices (physically) cut for drying tomatoes. Next, the pericarp was segmented manually with a brush tool for all the slices in the images

The mean values of the parameters ($T_{2},\;$μ-porosity) in function of the distance from the tomato cuticle were computed using the same masks same as the ones used for $T_{2}$ maps estimation.

Statistical Data, Experiment A: The mean of the parameters were measured using the same masks same as the ones used for $T_{2}$ maps estimation.

Statistical Data, Experiment B: On fresh tomatoes, the mean of the parameters were measured on regions of interest (ROIs), corresponding to the pericarp, delimited manually using ImageJ software;

On dried tomatoes (DD1, DD2, DD3) the mean of the parameters were measured using the same masks same as the ones used for $T_{2}\;$maps estimation.

### 4.4 Unilateral NMR

UNMR measurements were performed at 10 ± 1 °C, regulated by the laboratory air-conditioning.

#### 4.4.1 Acquisition

The full description of data acquisition, profiles and T2 relaxation curves, can be found in the associated research article [1]. Briefly, both measurements were performed using CPMG pulse sequence. For profiles, 256 echoes were acquired at each depth with the repetition time (TR) of 4 s and 4 averages. Nearly 80 100µm-slice thick depths and 200µ step were acquired for each profile with 92 µs of echo spacing. For T2 measurement, 2048 (for DD0 and DD2) or 1024 echoes were acquired to sample the traversal decays for at least 3 depths in the middle of the tomato slice using with the following parameters with 100µs of echo spacing, a TR of 20 s and 16 averages. These acquisition parameters are resumed in the metadata file named acqu.par located in the data folder of each sample (see file and folder hierarchy description).

#### 4.4.2 Signal processing

Profiles are reconstructed by averaging the 256 CPMG echoe decays recorded at each depth to produce the SI.

As described in the research article, T2 distribution at each analyzed depth was obtained by fitting the 2048 or 1024 echo decays to a multi-exponential model. The signal decays were first pre-processed by removing the first two echoes and those following the first negative amplitude value, i.e., noise. Inverse Laplace Transform was then performed using an in-house Matlab® 2022a (MathWorks, Natick, MA, USA) implementation of the non-negative least square (NNLS) inversion algorithm (35), fed with 100 T2 values logarithmically spaced from 0.3 ms to 1000 ms along with a regularization parameter aimed at balancing fit stabilization and bias. The fit result of each trial is provided.

### 4.5 Histology

As described in the associated research article [1], histological measurements were performed with macrovision device. Samples were dyed with a 1 ‰ diluted solution of Fluorescent Brightener 28 (also known as *Calcofluor White*) to induce fluorescence under 365 nm UV light on the cellulose (i.e. the cell wall).

All images were analyzed with FIJI. Because of the difficulty to slice the samples cleanly, especially at FRESH and DD3 drying states, either with vibratome or with a razor blade, cell sizes were estimated with unavoidable uncertainty. When possible, an automatic segmentation method (watershed from *MorphoLibJ* plugin) was applied on images to automatically detect cell wall, however most of the cell sizes were obtained through manual thresholding, varying as a function of the image file. Only one operator performed the analysis, in order to minimize the uncertainty due to manual segmentation.

### 4.6 Tissue texture

As described in the associated research article [1], the texture profile analysis consists of two successive compressions. These mimick the mastication and 13 variables (detailed in the R Markdown file) describing the textural behavior of food products are extracted from the test curve. These variables were extracted from Nexygen software and analysed with R Studio.

### 4.7 Chemical composition

The analysis protocol for carotenoids, polyphenols, sugars and acids are described in the associated research paper [1]. Upon quantification of the concentration of the molecules of interest, statistical analyses were performed with R Studio in order to identify the factors (cultivar, drying state) inducing variations of concentration. All analyses are presented in the R Markdown file and the dataset documentation Markdown file.

## Limitations

Not applicable.

## Ethics Statement

The authors have read and follow the ethical requirements for publication in Data in Brief and confirming that the current work does not involve human subjects, animal experiments, or any data collected from social media platforms.

## CRediT Author Statement

**Guylaine Collewet:** conceptualization, methodology, validation, investigation, writing, review & editing, data curation. **Amidou Traore:** conceptualization, methodology, validation, investigation, writing, review & editing, data curation. **Alexandre Leca:** conceptualization, methodology, validation, investigation, formal analysis, writing, review & editing, data curation. **Sylvain Challois:** investigation, data curation. **Clémentine Lorho:** investigation. **Gauvain Pierre:** investigation. **Nathan Rausch de Traubenberg:** investigation. **Yves Diascorn:** methodology, investigation. **Stéphane Quellec:** methodology, investigation. **Caroline Garcia:** investigation. **Carine Le Bourvellec:** conceptualization, investigation. **Valérie Serra:** resources. **Sylvie Clerjon:** conceptualization. **Marc Lahaye:** conceptualization, review & editing. **Nadia Bertin:** conceptualization, supervision of the experimental production of fruit, review & editing. **Maja Musse:** conceptualization, methodology, validation, investigation, writing, review & editing, data curation, supervision, project administration.

## Data Availability

https://recherche.data.gouv.frData for analyzing the drying process in tomatoes (Original data).

## References

[1] Musse M. (2025). Insights into the mechanisms involved in the evolution of the structural and physicochemical properties of tomato during air drying – a study combining MRI, unilateral NMR and conventional techniques. Food Res. Int..

[2] Collewet G. (2022). Multi-exponential MRI T2 maps: a tool to classify and characterize fruit tissues. Magn. Reson. Imaging.

[3] Musse M. (2010). Quantification of microporosity in fruit by MRI at various magnetic fields: comparison with X-ray microtomography. Magn. Reson. Imaging.

